# A self-assembling graphene oxide coating for enhanced bactericidal and osteogenic properties of poly-ether-ether-ketone

**DOI:** 10.3389/fbioe.2024.1378681

**Published:** 2024-05-06

**Authors:** Run Huang, Yingjian Gu, Yeju Yuan, Yunxiao Wang, Yusong Pan, Bo Li, Geliang Ren, Lei Huang, Yinghai Xie

**Affiliations:** ^1^ The First Affiliated Hospital of Anhui University of Science and Technology, Huainan, China; ^2^ School of Materials Science and Engineering, Anhui University of Science and Technology, Huainan, China; ^3^ Institute of Environment-Friendly Materials and Occupational Health of Anhui University of Science and Technology, Wuhu, China; ^4^ Medical School, Anhui University of Science and Technology, Huainan, China; ^5^ State-Key Laboratory for Mechanical Behavior of Materials, Xi’an Jiaotong University, Xi’an, China; ^6^ Department of Gastrointestinal Surgery, Hubei Cancer Hospital, Wuhan, China

**Keywords:** poly-ether-ether-ketone (PEEK), graphene oxide (GO), bactericidal effect, bioactivity, osteoblasts

## Abstract

Poly-ether-ether-ketone (PEEK) is a biomedical plastic that can be used for orthopedic implants, but it offers poor antibacterial properties and bioactivity. In this study, PEEK was sulfonated with the obtained porous structure adsorbing graphene oxide (GO). The surface microstructures and properties of the original PEEK, sulfonated PEEK (SPEEK), and GO-grafted PEEK (GO-SPEEK) were characterized. The results revealed that the GO-SPEEK surface is a 3D porous structure exhibiting superior hydrophilicity to the original PEEK. Although SPEEK was shown to possess antimicrobial properties against both *Escherichia coli* and *Staphylococcus aureus*, the bactericidal effect was even more significant for GO-SPEEK, at about 86% and 94%, respectively. In addition, the *in vitro* simulated-body-fluid immersion and cell experiments indicated that GO-SPEEK had much better hydroxyapatite (HA)-precipitation induction capacity and cell–material interactions (e.g., cell adhesion, proliferation, osteodifferentiation, and extracellular matrix mineralization. The tensile test revealed that the mechanical properties of PEEK were maintained after surface modification, as GO-SPEEK has comparable values of elastic modulus and tensile strength to PEEK. Our investigation sought a method to simultaneously endow PEEK with both good antimicrobial properties and bioactivity as well as mechanical properties, providing a theoretical basis for developing high-performance orthopedic implants in the clinic.

## Graphical Abstract

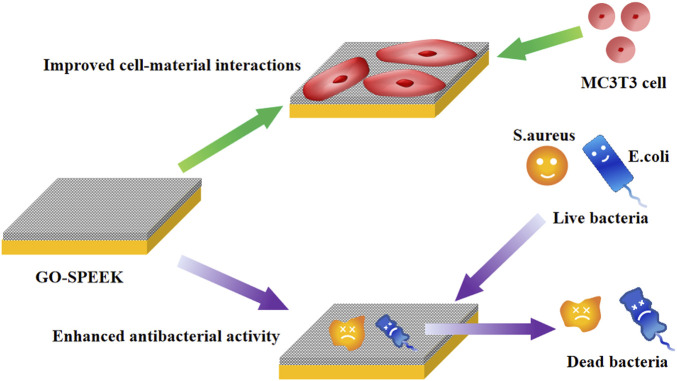

## 1 Introduction

Poly-ether-ether-ketone (PEEK) is a new and special engineering thermoplastic that has attracted extensive attention because of its huge potential for orthopedic implants (Basgul et al., 2018). In comparison to traditional metallic materials with high elastic modulus, such as titanium and 316L stainless steel, PEEK has been proven to have a lower elastic modulus (<6 GPa) close to that of human bone (Lee et al., 2012), which could reduce the possibility of bone resorption and osteoporosis caused by stress shielding (Lu et al., 2014; Kurtz, 2019), thus improving the success of orthopedic implant surgery. It also has the properties of good biocompatibility, chemical inertness, and low cytotoxicity and does not cause inflammation or other pathological issues for the patient (Stratton-Powell et al., 2016; Liu et al., 2017) while implanted into the human body. Additionally, the radiation permeability of PEEK means that it does not interfere with computed tomography (CT) and magnetic resonance imaging (MRI) techniques commonly used in medicine, allowing these techniques to monitor bone growth and healing after surgery (Toth et al., 2006; Sobieraj et al., 2009). However, the poor antibacterial properties and bioactivity of PEEK limit its wider application in the orthopedic field (Liu et al., 2017; Mo et al., 2020).

In recent years, researchers have used a series of surface modification approaches to improve the antimicrobial properties and bioactivity of PEEK. Liu et al. (2019) introduced nano-copper ions on the surface of sulfonated PEEK by ion implantation, which showed resistance to methicillin-resistant *Staphylococcus aureus* in an *in vitro* antibacterial assay. Additionally, it was reported that the O_2_/Ar or NH_4_ plasma-treated PEEK surfaces could enhance the adhesion, proliferation, and osteogenic differentiation of adipose mesenchymal stem cells compared to the original PEEK (Waser-Althaus et al., 2014). Some researchers deposited hydroxyapatite layers on the surface of PEEK by plasma spray coating, vacuum-plasma-spray, or electrophoretic deposition and found the surface-modified PEEK dramatically improved the bioactivity (Lee et al., 2013; Mahjoubi et al., 2017; Bastan et al., 2018). However, these surface treatment methods, such as ion implantation, were reported to be much more energetic and easily injured the surface of polymers such as PEEK (Popok, 2019). Although the method of thermal spraying could endow the PEEK surface with a good biological effect, the prepared coating was usually reported to possess weak bonding with the PEEK substrate and easily peeled off (Ma et al., 2023).

Recently, an *in situ* modified method, sulfation treatment, has attracted research attention because it could fabricate a three-dimensional porous structure on the PEEK surface, providing a suitable site for cell adhesion, enhancing cell proliferation, and promoting the development of vascularization as well as bone tissue growth (Deng et al., 2015; Naskar et al., 2017; Bastan et al., 2018). In addition, the sulfation-achieved porous structure has been found to enhance the interfacial combination of late adsorption factors/molecules and was recognized as a good loading platform for PEEK (Ouyang et al., 2018a; Sun et al., 2018).

Graphene oxide (GO) is an oxidized form of graphene, a new two-dimensional carbon material with many oxygen-containing functional groups on its surface, such as hydroxyl (C-OH), carboxylic (-COOH), carbonyl (C=O), and epoxide groups (C-O-C), which enables it to have excellent hydrophilic properties (Kiew et al., 2016). GO exhibits excellent antibacterial properties against Gram-negative and Gram-positive bacteria (He et al., 2015) and is a good candidate for fighting bacterial infections because it is free from the issue of antibiotic resistance. In addition, some scholars have noted that GO has both excellent antibacterial properties and positive regulation of cellular activity, and it could promote the attachment, proliferation, and osteogenic differentiation of bone marrow mesenchymal stem cells (BMSCs) due to the ability of its surface hydrophilic functional groups to regulate cell behavior and protein adsorption (Hong et al., 2012; Luo et al., 2015).

Enlightened by the aforementioned research, zinc oxide/GO (Yang et al., 2022), MnFe_2_O_4_/GO (Yang et al., 2024), and nisin/GO (Kumar et al., 2023) systems were respectively designed and deposited onto the sulfonated PEEK. However, the composites exerted the antibacterial effect in a mass-ratio-dependent manner. Single GO was coated onto the sulfonated PEEK by Guo et al. (2021a) and Ouyang et al. (2018b) to explore the biological effect of the modified surfaces, either dental pathogens (*P. gingivalis* and *S. mutans*) or tumor-derived cell lineage (MG-63). However, the variations in the mechanical properties of PEEK after the surface treatments were neglected. In fact, adequate mechanical strength is crucial for the service of a biomaterial (Qian et al., 2024a).

In this study, we first prepared a porous surface on PEEK by sulfonation and then grafted GO onto the sulfonated surface to explore how the modified surface influences the growth of bacteria [Gram-negative *Escherichia coli* and Gram-positive *Staphylococcus aureus* bacteria; these two strains are the main pathogenic microbes responsible for implant-related infections (Qian et al., 2024b)] and the osteogenic properties of cells [MC3T3-E1, a pre-osteoblast cell lineage; its ability to differentiate to osteoblasts is considered a precursor for the successful osteointegration of an implant (Bhaskar et al., 2023)]. In addition, the mechanical properties of the untreated/treated PEEK were also compared. Our obtained results identified that PEEK could be modified by simply combining treatment with surface sulfonation and GO grafting to simultaneously gain both good antimicrobial properties and bioactivity as well as mechanical properties, laying a theoretical foundation for the clinical development of long-lasting orthopedic implants.

## 2 Experimental methods

### 2.1 Materials

Pure PEEK (99%) was purchased from Junhua Technology (Changzhou, China). GO liquid dispersion with a concentration of 1 mg/mL was provided by Dazhan NAMI (Guangdong, China). Concentrated sulfuric acid, acetone, and ethanol were obtained from Kolon Chemical (Chengdu, China).

### 2.2 Sample preparation

Pure PEEK was crushed into a 10 mm × 10 mm × 3 mm sheet. The sheets were successively polished with 200-, 300-, 500-, 800-, 1,200-, 2000-, 3,000-, 5,000-, and 7000-grit silicon carbide sandpaper and then sequentially put into acetone, anhydrous ethanol, and ultra-pure water for ultrasonic cleaning for 15 min, respectively, to remove impurities from the surfaces.

**FIGURE 1:**
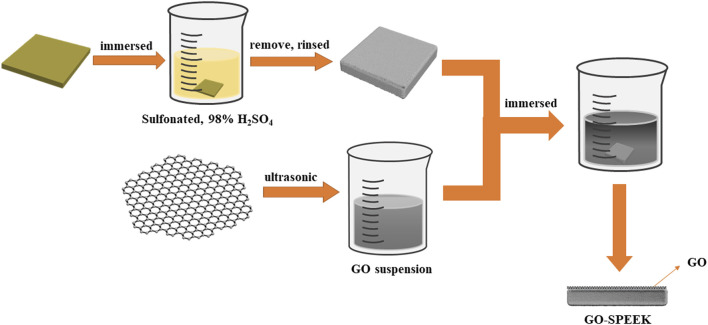
The preparation procedure for the GO-SPEEK sample.The shape of the sample for the tensile test.

The cleaned PEEK sheets were immersed into a solution of sulfuric acid at a concentration of 95 wt% for 5 min at 25°C and then washed in a 1 mol/L NaOH solution to remove the residual sulfuric acid. The above sheets were then dried in an oven at 60°C. Afterward, the sulfonated PEEK pieces were designated as SPEEK. The SPEEK specimens further modified with GO (designated as GO-SPEEK) were prepared as follows: first, the SPEEK specimens were immersed in a GO dispersion at a concentration of 1 mg/mL for 5 min, and then the specimens were removed and dried in an oven at 60°C for 30 min. The schematic diagram of the GO-SPEEK sample preparation procedure is shown in Figure 1.

### 2.3 Surface structure characterization

Scanning electron microscopy (SEM, Zeiss Gemini 300, Germany) was used to observe the surface morphology of PEEK, SPEEK, and GO-SPEEK. All samples were sprayed with gold for 2 min before the SEM observation with a scanning wavelength range of 3,000–500 cm^−1^. Fourier transform infrared spectroscopy (FT-IR, Hermo Scientific Nicolet iS20, America) was used to analyze the surface chemical composition of the samples by using attenuated total reflection. The crystal state of samples was tested by X-ray diffraction (XRD, Rigaku Smartlab SE, Japan) with a scanning range of 2θ from 10° to 80° and a step length of 5°/min. The Raman spectra of the different samples were recorded using the LabRam HR Evolution (SPM-960, Japan) laser Raman spectrometer, with a laser wavelength of 633 nm and a wavenumber range of 2,500–1,000 cm^−1^. In addition, X-ray photoelectron spectroscopy (XPS, Thermo Scientific ESCALAB, United States) with Al Kα radiation was utilized to detect the chemical composition and elemental state of the elements.

### 2.4 Surface wettability of the samples

The hydrophilicity of the samples was assessed by the static droplet method using a contact angle tester. The volume of water droplets was 4 μL. After the droplet touched the surface of the samples for 5 s, the water contact angle was recorded. Three parallel samples were selected for each group, and three different locations on the surface of each sample were tested.

### 2.5 Antibacterial test

The antimicrobial capacity of the samples was assessed by using the bacterial strains of *Escherichia coli* (*E. coli*, ATCC25922) and *Staphylococcus aureus* (*S. aureus*, ATCC25923). The bacterial inoculum was prepared with 10 g/L peptone, 5 g/L NaCl, and 3 g/L beef paste. *E. coli* and *S. aureus* were then added to PBS at a concentration of 10^6^ cells/mL (calibrated according to colony forming units (CFU) using the diffusion plate method). The tested samples were sterilized using medical alcohol immersion as well as UV irradiation and then placed in individual wells of a 24-well culture plate. A 1-mL aliquot of the above bacterial suspension was added to each sample in the wells and incubated at 37°C for 24 h. To examine the bacterial morphology, the samples were washed three times with PBS to remove the unadhered bacteria and immersed in 2.5% glutaraldehyde for 1 h at 4°C. The samples were subsequently dehydrated in ethanol and then vacuum-dried. After gold spraying, the samples were observed under FESEM. To quantitatively explore the live bacteria colonies on different surfaces, the above-mentioned 24-h incubation samples were rinsed using PBS and placed separately in 3 mL of PBS. After being ultrasonically shaken to detach the adhered bacteria from the samples, the resulting bacterial suspension was diluted 10,000-fold and incubated at 37°C for 24 h in a standard agar medium (Huang et al., 2021), after which the active bacteria were counted by the plate method. Three samples for each group were tested, and each test was repeated three times (*n* = 3).

### 2.6 Simulated body fluid (SBF) immersion

The biomineralization capacity of different samples was assessed by exploring the hydroxyapatite (HA)-precipitation activity on the surfaces by immersing the samples in a simulated body fluid (SBF) solution. The SBF solution was formulated as follows: NaCl (8.035 g), NaHCO_3_ (0.355 g), KCl (0.255 g), K_2_HPO_4_ (0.176 g), MgCl_2_ (0.146 g), CaCl_2_ (0.292 g), and Na_2_SO_4_ (0.072 g). During the preparation, the ingredients were dissolved in deionized water and kept at a temperature of 36.5°C, and the solution was adjusted to pH = 7.35 by using 1 mol/L hydrochloric acid and Tris (hydroxymethyl)aminomethane (Tris) (Barooghi et al., 2018). The samples were placed in centrifuge tubes after the SBF solution was added, and the tubes were transferred into a constant temperature incubator at 37°C. At the target times of 14 days and 28 days, the samples were removed, dried, and sprayed with gold to observe the surface morphology by SEM. In addition, to reveal the surface elemental distribution of the SBF-immersed samples, EDS-mapping analysis was conducted on the SEM equipment using an SEM-EDS system. The substances formed on the different surfaces were also investigated by means of XRD.

### 2.7 *In vitro* cell response experiment

#### 2.7.1 Cell culture

The mouse embryonic osteoblastic cell line, MC3T3-E1, was purchased from the Institute of Biochemistry and Cell Biology of the Chinese Academy of Sciences (Shanghai, China). The cells were cultured in normal growth medium (DMEM; HyClone, United States) supplemented with 10% fetal bovine serum (Gibco; Thermo Fisher Scientific, United States) and 1% penicillin/streptomycin (Sigma, United States). Cells were used for four passages and incubated in an atmosphere with the CO_2_ of 5% and the air of 95% at a temperature of 37°C. Cells were seeded on different sample surfaces in 24-well plates at a density of 5×10^4^ cells per well, and the culture medium was refreshed every 2 days.

#### 2.7.2 Cell morphology, cytotoxicity, and cell proliferation

To explore the cell adhesion on different sample surfaces, after 5 h of culture, the cell-adhered samples were gently rinsed three times with PBS to remove the unadhered cells. Then, the samples were immersed in a 2.5% glutaraldehyde solution for 1 h. After that, the cell-fixed samples were dehydrated with a gradient concentration of ethanol for 15 min at every step. Finally, the samples were dried and sprayed with gold, and the typical morphology of the cells on the samples was observed by SEM. The cytotoxicity and proliferation of cells on different sample surfaces were evaluated using a Cell counting kit-8 (CCK-8) assay (Qian et al., 2023). Specifically, the samples were laid centrally in 24-well plates, and cells were seeded on each sample and incubated for 1 h, 5 h, 24 h, 72 h, 168 h, and 336 h. At each targeted time, the complete medium was discarded, and the samples were washed three times with PBS and then transferred to new 24-well plates. Then, 1 mL of medium containing 100 mL of CCK-8 reagent (Dojindo, Japan) was added to each well and incubated for a further 4 h in the incubator. Afterward, the cell viability of each sample was measured at an absorbance of 450 nm using a microplate reader. Three replicates for each group were tested, and each test was repeated three times (*n* = 3).

#### 2.7.3 Osteogenesis-related gene expressions

The expression of osteogenesis-related genes (such as RUNX2, OCN, and COL-I) in cells cultured on the different samples was analyzed by real-time polymerase chain reaction (real-time PCR). After incubation for 3 days, 7 days, and 14 days, the total RNA from the samples was extracted using an RNA kit (Gibco, United States). Then, 1 μg of RNA from the cells on each sample was reversely transcribed into complementary DNA using a PrimeScripTM RT reagent kit (TaKaRa, Japan). Subsequently, the expression of osteogenesis-related genes was quantified via an RT-PCR method, as described in detail in our previous work (Huang et al., 2021). Genes and related specific primers are listed in Table 1, and the acquired relative expression data were all normalized against glyceraldehyde-3-phosphate dehydrogenase (GAPDH) using the delta Ct method. Three samples from each group were tested, and each test was repeated three times (*n* = 3).

**TABLE 1:** The specific primer set sequences.

Target	Primer sequences
RUNX2	Forward primer: 5′-TGG​TGT​TGA​CGC​TGA​TGG​AA-3′
	Reverse primer: 5′-ATA​CCG​CTG​GAC​CAC​TGT​TG-3′
OCN	Forward primer: 5′-CTT​CGT​GTC​CAA​GAG​GGA​GC-3′
	Reverse primer: 5′-CAG​GGG​ATC​CGG​GTA​AGG​A-3′
Col-I	Forward primer: 5′-TGC​AGG​GCT​CCA​ATG​ATG​TT-3′
	Reverse primer: 5′-AGG​AAG​GGC​AAA​CGA​GAT​GG-3′
GAPDH	Forward primer: 5′-ATC​AAG​TGG​GGT​GAT​GCT​GG-3′
	Reverse primer: 5′-TAC​TTC​TCG​TGG​TTC​ACG​CC-3′

#### 2.7.4 Evaluation of the extracellular matrix (ECM) mineralization property

Alizarin red staining (ARS) was used to assess the ECM mineralization of the cells cultured on different samples. After 7 days and 14 days of culture, the cell-seeded samples were fixed in 4% paraformaldehyde for 30 min. Then, the samples were stained with ARS solution (2%, pH 4.2; Sigma-Aldrich) for 20 min. After washing the stained samples with deionized water several times to remove the excess ARS, the stained pictures of the samples were taken with an optical microscope. To quantitatively reveal the ECM mineralization of the samples, the stained samples were also immersed into hexadecyl pyridinium chloride (1 w/v%; Sigma-Aldrich) and shaken for 2 h. The absorbance values were measured at 550 nm. Three samples for each group were tested, and each test was repeated three times (*n* = 3).

#### 2.7.5 Statistical analysis

The obtained data from the above cell experiments were analyzed by SPSS 14.0 software and expressed as the mean ± standard deviation (SD) for *n* = 3. Statistically significant differences (p) between the groups were detected by a Student’s t-test. *p* < 0.05 was considered statistically significant, and *p* < 0.01 was considered highly statistically significant.

### 2.8 Mechanical properties

**FIGURE 2:**
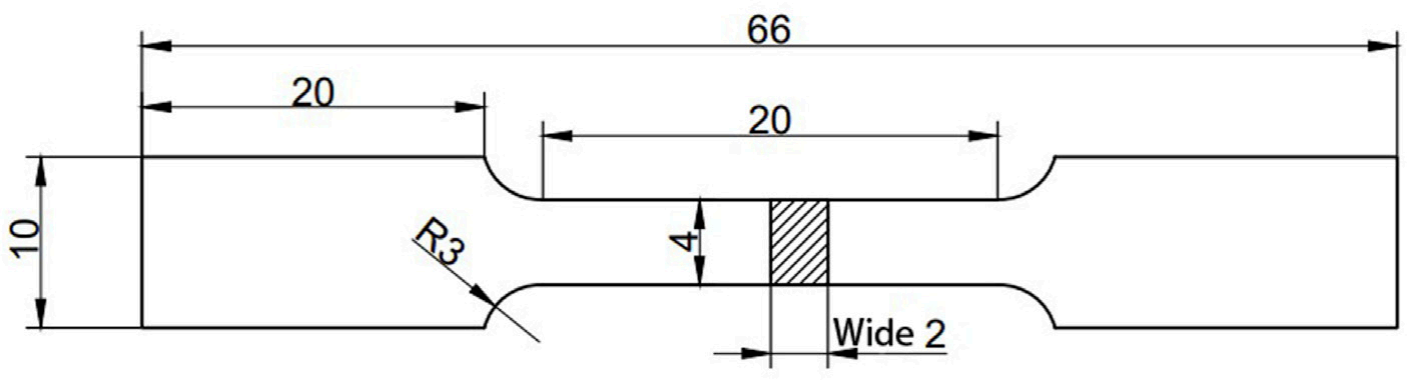
The shape of the sample for the tensile test.

To display the effect of surface modification on the mechanical properties of PEEK, a tensile test was conducted on the samples using an electronic universal testing machine (TY8000-A) in accordance with the ASTM standard D638 (Laureto and Pearce, 2018; Zhao et al., 2023). The shape of the test sample is shown in Figure 2. Three samples for each group were used to obtain the average.

## 3 Results and discussion

### 3.1 Surface characterization of the samples

**FIGURE 3:**
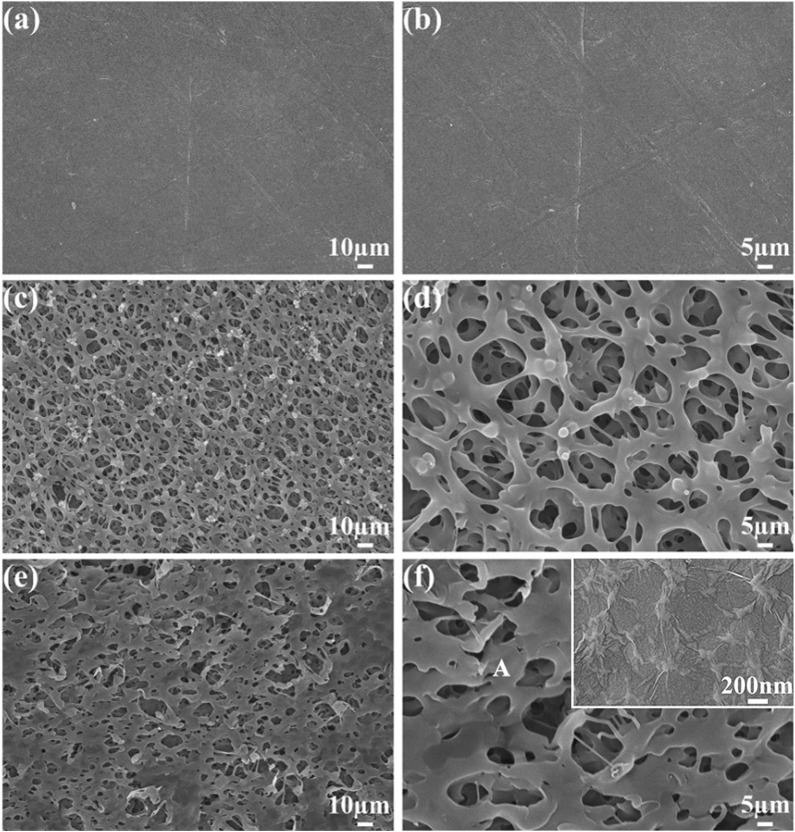
SEM images show the surface morphology of **(A, B)** PEEK, **(C, D)** SPEEK, and **(E, F)** GO-SPEEK. The inset in the upper right corner of (F) is the magnified view of the location in (F) indicated with the letter A.

Figure 3 shows the PEEK, SPEEK, and GO-SPEEK surface morphology. As shown in Figures 3A, B, after being ground and polished, the PEEK surface is smooth and flat, Figures 3C, D show the SPEEK surface morphology; a three-dimensional (3D) porous structure with an average pore size of about 8 μm is formed on the surface. After further coating with graphene oxide, the porous surface varies compared to the sulfonated surface. Part of the 3D porous structure is covered by the GO layer, leading to a decrease in the average pore size (Figures 3E, F). To further disclose the morphology of the grafted GO, a magnified image (the inset in the upper right corner of Figure 3F) is taken at the location indicated with the letter A; it demonstrates that the GO deposited on the SPEEK sample exhibits a wrinkled-like sheet appearance, which is similar to the results reported by Kumar et al. (2023).

**FIGURE 4:**
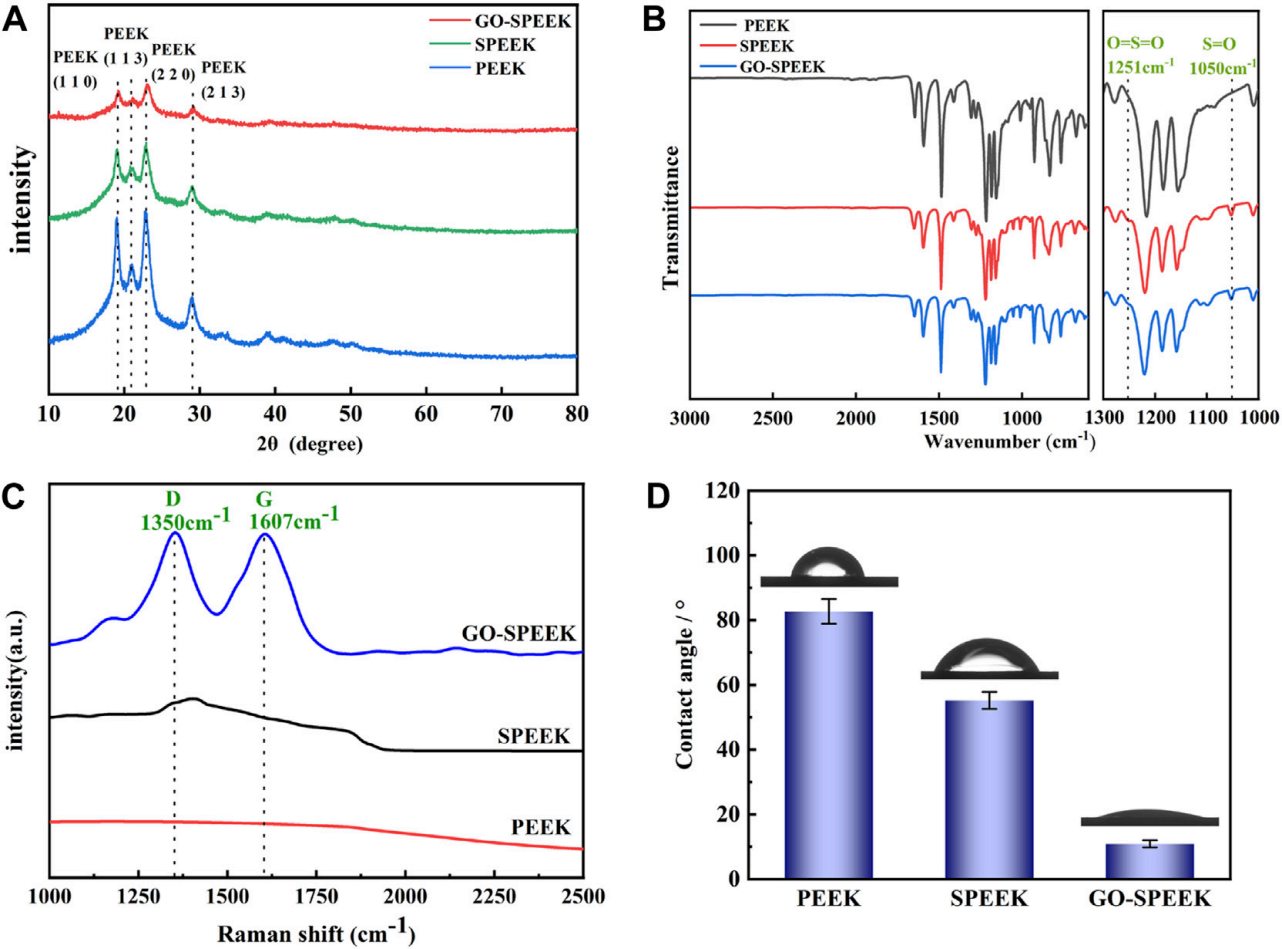
Surface characterization of different samples: **(A)** XRD patterns, **(B)** FTIR spectra, **(C)** Raman spectra, and **(D)** the surface water contact angle of PEEK, SPEEK, and GO-SPEEK; data are given as the mean ± SD (*n* = 3).

Figure 4A depicts the XRD crystal phase of the different samples. As can be seen, PEEK is a semi-crystalline polymer exhibiting relatively sharp crystalline peaks, with higher-intensity peaks at 2θ about 19°, 21°, 24°, and 29°, corresponding to the diffraction of (110), (113), (220), and (213) crystalline planes respectively, which is similar to the previously reported results (Cao et al., 1994). SPEEK and GO-SPEEK also have characteristic peaks at the same positions with weakened intensity, indicating that the surfaces of the samples have been modified. The FTIR spectra of different samples are shown in Figure 4B. SPEEK and GO-SPEEK show new absorption peaks at 1,050 cm^−1^ and 1,251 cm^−1^. The 1,050 cm^−1^ peak corresponds to the S=O symmetric stretching absorption peak, and the 1,251 cm^−1^ peak corresponds to the O=S=O asymmetric stretching absorption peak (Zhao et al., 2013; Chen et al., 2018). This indicates that the -SO_3_H functional group has been introduced into the PEEK polymer chain by the sulfonation reaction (Zhao et al., 2013). Because of the adsorption effect of the micropores generated by the sulfonation, GO is confirmed as being successfully loaded on the SPEEK surface. As shown in Figure 4C, GO-SPEEK has two characteristic peaks that appear around 1,350 cm^−1^ and 1,607 cm^−1^, corresponding to the D-band and G-peaks (Ferrari and Basko, 2013). The peaks represent defects in the C-atom lattice and in-plane stretching vibrations of the sp2-hybridized C-atoms, respectively.

It was reported that the biological properties of the biomaterials are closely related to their surface hydrophilicity (Wang et al., 2019; Kopac, 2021). Therefore, a water contact angle tester was used to analyze the hydrophilicity of different samples. The results in Figure 4D reveal that the water contact angle of the original PEEK surface is 82.7° ± 3.8°, and the value decreases to 55.2° ± 2.6° for SPEEK due to the introduction of the more hydrophilic -SO_3_H by the electrophilic substitution reaction (Alimohammadi and Ramazani, 2023). Introducing the GO on the SPEEK surface further reduces the water contact angle to 10.9° ± 1.1°, which might be due to the hydrophilic functional groups, such as hydroxyl and carboxyl groups, on the modified surface (Dikin et al., 2007), causing further improvement of the GO-SPEEK hydrophilicity.

**FIGURE 5:**
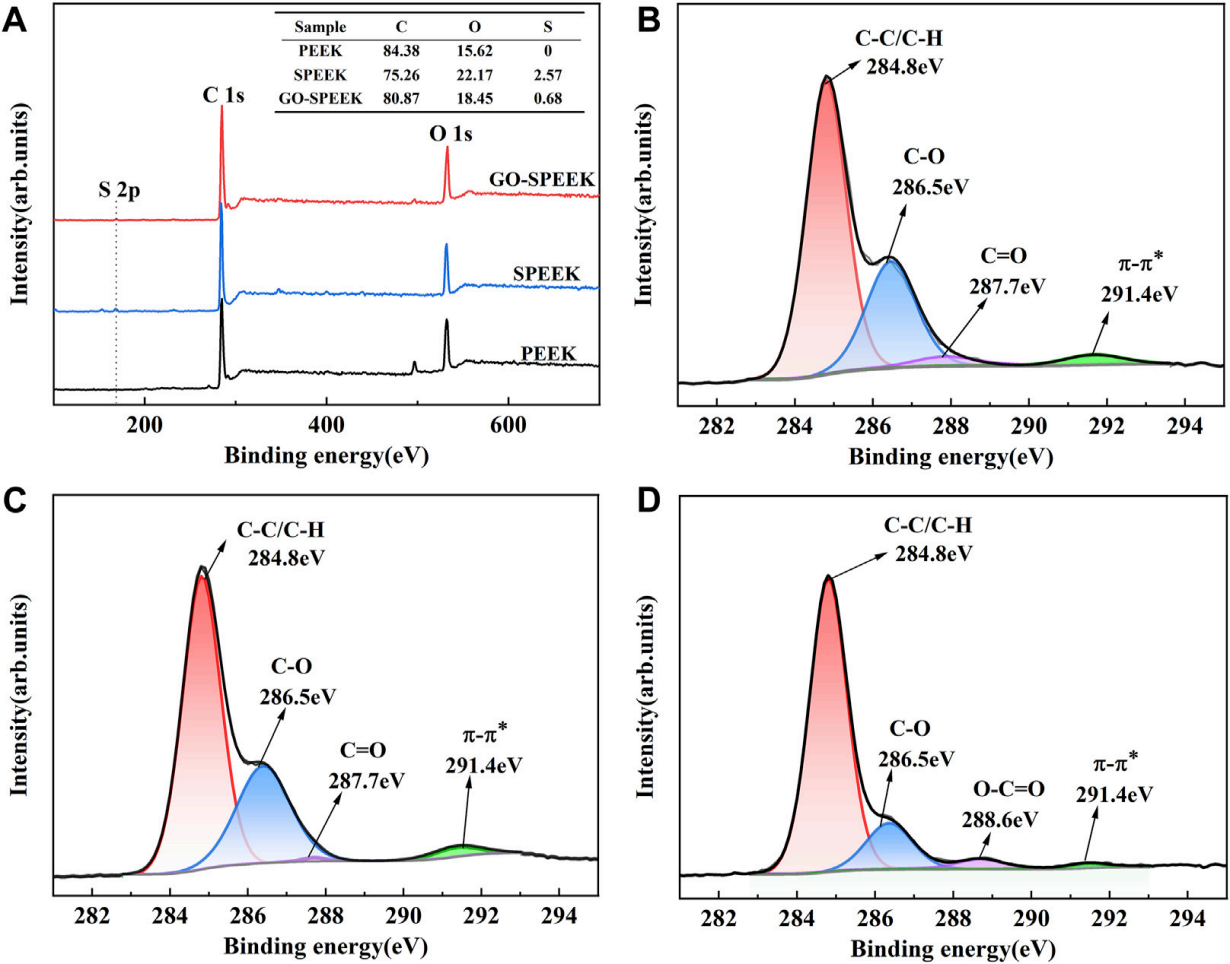
**(A)** XPS wide-scan spectra of different samples and high-resolution C 1s XPS spectra of **(B)** PEEK, **(C)** SPEEK, and **(D)** GO-SPEEK.

XPS was adopted to explore the chemical composition before and after surface treatment. The wide-scan spectra show that only C and O are detected for the PEEK surface. A minor amount of S could be detected for the SPEEK surface, and the relative S percentages of PEEK, SPEEK, and GO-SPEEK are 0%, 2.57%, and 0.68%, respectively (Figure 5A), suggesting a significant decrease of S after being grafted with GO. The high-resolution spectra of C 1s of different samples are shown in Figures 5B–D. The characteristic peaks appearing at 284.8 eV and 286.5 eV correspond to the C-C/C-H and C-O groups, respectively (Ouyang et al., 2018b; Al-Gaashani et al., 2019). C=O bond and π-π* bonds at 287.7 eV and 291.4 eV are observed for PEEK (Figure 5B, which change little after sulfonation (Figure 5C), similar to previous reports in the literature (Ouyang et al., 2018b). However, an O-C=O bond appears for GO-SPEEK (Figure 5D), suggesting that GO is successfully loaded on the SPEEK surface (Torrisi et al., 2020). This can also be evidenced by the increased C content of GO-SPEEK compared to SPEEK (Figure 5A) (Al-Gaashani et al., 2019).

### 3.2 Antibacterial properties

**FIGURE 6:**
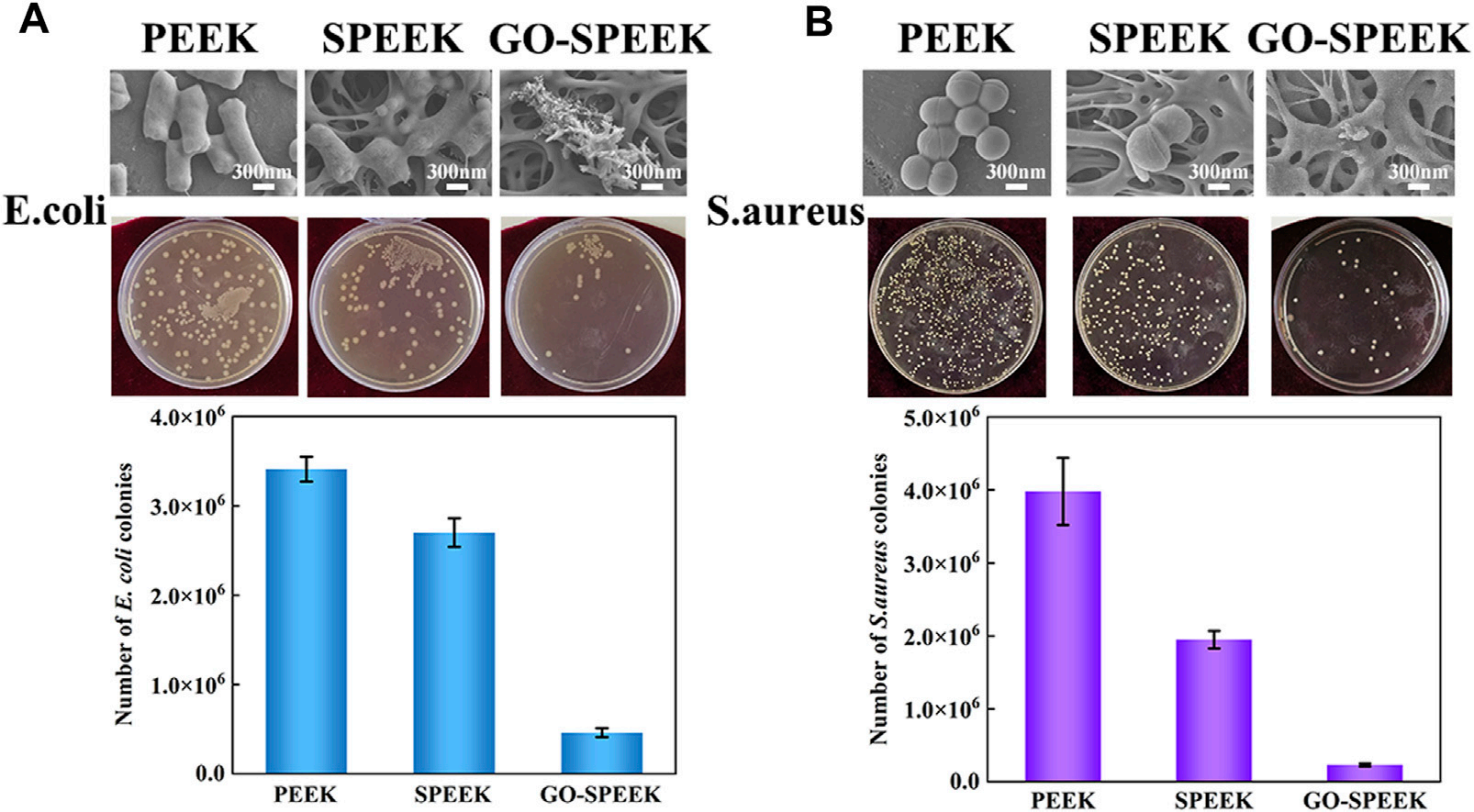
*In vitro* antibacterial property evaluation. **(A)** SEM images of *Escherichia coli* after 24-h incubation on PEEK, SPEEK, and GO-SPEEK, agar plate culture pictures, and the number of *Escherichia coli* colonies on the different samples. **(B)** SEM images of S. aureus after 24-h incubation on different surfaces, the agar plate culture pictures, and the number of *S. aureus* colonies on the samples. Data are given as the mean ± SD (*n* = 3).

It has been previously observed that there is a significant competitive surface adhesion relationship between the invading bacteria and the host cells, which is described as surface competition (Gristina, 1987). Free bacteria adhere to the implant surface and subsequently form a stubborn biofilm that disrupts host-cell adhesion, leading to a failure of implant-cell integration. It is essential to prevent biofilm formation to avoid bacterial infections (Hemmati et al., 2021). Figure 6A shows the antibacterial effect of different samples on *E. coli*. As can be seen from the SEM result, after 24 h of co-culture, the PEEK surface shows the highest number of *E. coli*, and the bacteria exhibit a typically rod-like morphology. The profile of the bacteria is clear, and the cell membrane is relatively smooth. In contrast, the *E. coli* on the SPEEK surface reveals a deformed and elongated shape, suggesting that the SPEEK sample creates an uncomfortable environment for bacteria to live. Note that few *E. coli* could survive on the GO-SPEEK surface, and the attached *E. coli* displays a destructed appearance. The agar dilution method proves the above SEM result; the number of *E. coli* on the samples follows the order of PEEK > SPEEK > GO-SPEEK. The plate count result is shown in the histogram and indicates that the PEEK surface has the highest number of *E. coli* colonies at 3.45×10^6^, higher than the 2.72×10^6^ colonies formed on the SPEEK surface, and much higher than the 4.6×10^5^ colonies formed on the GO-SPEEK surface. It is evident that the GO-grafted PEEK surface has a significant bactericidal effect on *E. coli*; the number of *E. coli* is reduced by approximately 86% after the PEEK surface modifications.

*S. aureus* is one of the leading causes of hospital- and community-associated infections (Huang et al., 2011) and is a large proportion of the bacteria that infect orthopedic implants (Arciola et al., 2018). Therefore, it is even more important to examine the resistance of orthopedic implants to *S. aureus*. Figure 6B shows the antibacterial properties of different samples against *S. aureus*. The SEM results show that the *S. aureus* grows well on the PEEK surface and exhibits the typical morphology of grape bunches. Fewer *S. aureus* are observed on the SPEEK surface, but they still maintain the shape of intact balls. However, the *S. aureus* on the GO-SPEEK surface seems lysed and barely visible. The SEM result is highly consistent with the agar plate culture result: fewer *S. aureus* colonies are on the SPEEK surface, and many fewer colonies are on the GO-SPEEK surface than the PEEK surface. The quantitatively counted result reveals that the PEEK surface has the largest number of *S. aureus* colonies, 3.98×10^6^. The number of colonies decreases to 1.95×10^6^ for the SPEEK surface (a decline of approximately 51%), while the GO-SPEEK surface is observed to be more resistant to *S. aureus* with an approximately 94% reduction of bacteria colonies to about 2.3×10^5^. The grafted GO plays an important role in endowing GO-SPEEK with high efficiency in killing both Gram-negative and Gram-positive bacteria.

The grafted GO might greatly improve the antimicrobial effect of PEEK in two ways: first, the wrinkled GO nanosheet could provide many sharp edges (Ouyang et al., 2018b; Kumar et al., 2023), which can cause physical damage to the bacterial cytosol and destroy the bacterial membrane, thus causing cytoplasmic loss and ultimately leading to death of bacteria (Akhavan and Ghaderi, 2010). Second, the bacterial membrane is mainly composed of phospholipid macromolecules, and GO has a unique two-dimensional structure with sp^2^ carbon, resulting in strong dispersion between GO and phospholipids, which allows GO to continuously capture the phospholipids of the bacterial membrane and leads to the eventual destruction of the bacterial membrane (Tu et al., 2013). Some scholars have noted that the hydrophobic interactions between GO and phospholipids would contribute to bacterial phospholipid translocation and overturn of the cytosolic membrane and, therefore, exacerbate damage to the bacterial membrane (Dallavalle et al., 2015; Guo et al., 2021b). In this work, the surface-modified PEEK materials exhibit higher bactericidal capacity against the Gram-positive *S. aureus* than the Gram-negative *E. coli*. The reason may be that the two kinds of bacteria have different acid resistance. *E. coli* has the ability to transfer Gln to Glu, which produces gaseous ammonia. The free ammonia could neutralize protons, leading to an increase in intracellular pH and improving their resistance to acidic environments (Lu et al., 2013). In contrast, *S. aureus* is less resistant to acids. In our article, the sulfonation treatment introduced -SO_3_H groups, which are highly acidic, on PEEK (Sutherland et al., 1994; Ouyang et al., 2016). Therefore, the surface-modified PEEK shows much stronger antibacterial activity for the *S. aureus* strain.

### 3.3 Biomineralization capacity in SBF solution

**FIGURE 7:**
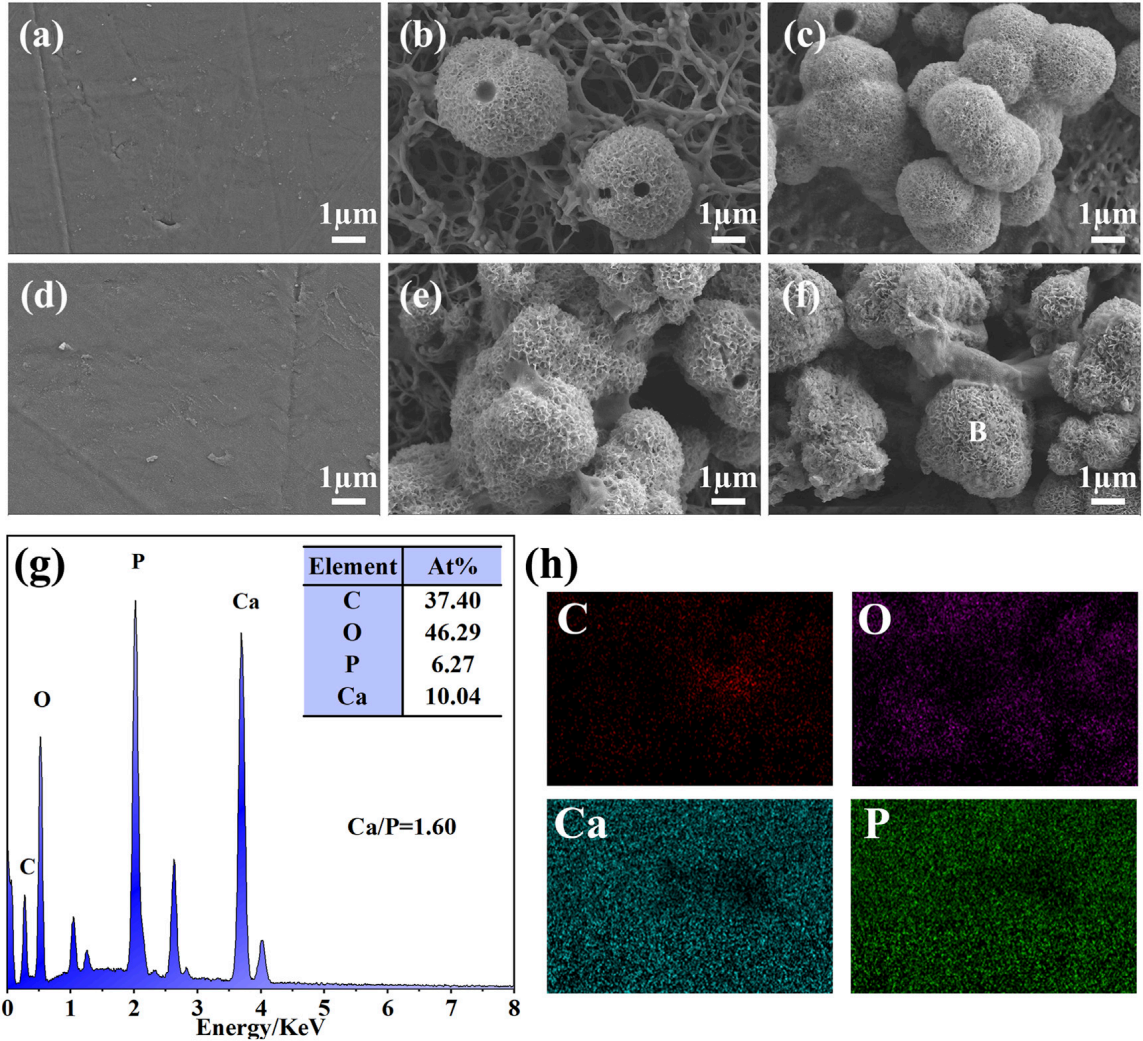
Surface morphology of different samples after soaking in SBF solution for 14 days: **(A)** PEEK, **(B)** SPEEK, and **(C)** GO-SPEEK; and after 28 days: **(D)** PEEK, **(E)** SPEEK, and **(F)** GO-SPEEK; **(G)** the EDS profile and elemental composition of the location indicated with a letter B in **(F)**; **(H)** EDS-mapping results showing the distribution of the C, O, Ca, and P in **(F)**.

**FIGURE 8:**
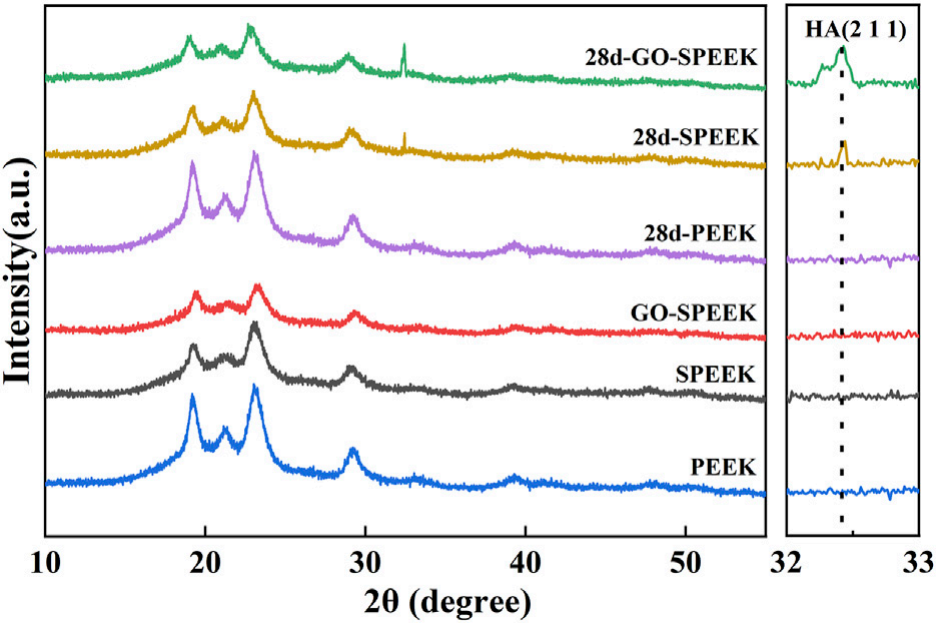
XRD patterns of PEEK, SPEEK, and GO-SPEEK before and after being immersed in SBF for 28 days.

The bioactivity of the biomaterials could be assessed by evaluating the ability to deposit HA on the surface after a certain time of *in vitro* SBF immersion. PEEK, SPEEK, and GO-SPEEK were placed in an SBF solution and incubated at 37°C in a constant temperature incubator. After 14 days and 28 days of immersion, the samples were dried and observed by SEM. As shown in Figures 7A, D, there is no morphological change on the PEEK surface after soaking for 14 days and 28 days, and no deposited material is observed on the PEEK surface. SPEEK, after 14 days of immersion, displays distinct ball-like deposits on the surface (Figure 7B), and the deposits become more dense after 28 days (Figure 7E). As for GO-SPEEK, the surface is almost totally covered by the ball-like deposits, even after 14 days (Figure 7C). The GO-SPEEK surface is completely covered by densely white precipitations after 28 days of immersion in the SBF solution (Figure 7F). The EDS results from a typical ball-like deposit (indicated with a letter B in Figure 7F) on the surface of GO-SPEEK after 28 days of immersion in SBF suggests that these balls are abundant in calcium (Ca) and phosphorus (P), and the Ca/P ratio is about 1.60 (Figure 7G), which is very close to the Ca/P ratio in HA (1.67). Figure 7H shows the EDS two-dimensional mapping result of Figure 7F. It can be seen that C, O, Ca, and P are generally evenly distributed throughout the surface. The Ca and P distributions are highly correlated, further indicating that Ca combines with P to form Ca/P precipitations on the GO-SPEEK surface. The XRD profiles of the samples before and after SBF immersion for 28 days are depicted in Figure 8. As can be seen, apart from the featured peaks belonging to PEEK, the peaks located at 2θ ≈ 32.4° appear on the surface-modified samples, which fit well to the (211) crystalline plane of HA (Almasi et al., 2014). These results indicate that sulfonation could enhance the biomineralization capacity of PEEK, while the introduction of GO could further boost the formation of HA on the SPEEK surface. GO-SPEEK exhibits a much stronger biomineralization capacity than the PEEK or SPEEK samples.

The sulfonation could introduce -SO_3_H groups on the samples, which was reported to enable ion exchange and electrostatic interaction of ions with the SBF solution, thus favoring surface mimetic mineralization (Hamai et al., 2018). In addition, it has been reported that the negatively charged GO surface could adsorb a significant amount of positively charged calcium ions from the SBF solution, which facilitates the combination of the PO_4_^3−^ ions and subsequently accelerates the precipitation of HA in the mineralization process (Gao et al., 2015). Accordingly, the biomineralization capacity of the investigated samples in this work is GO-PEEK > SPEEK > PEEK.

### 3.4 Cell behavior

**FIGURE 9:**
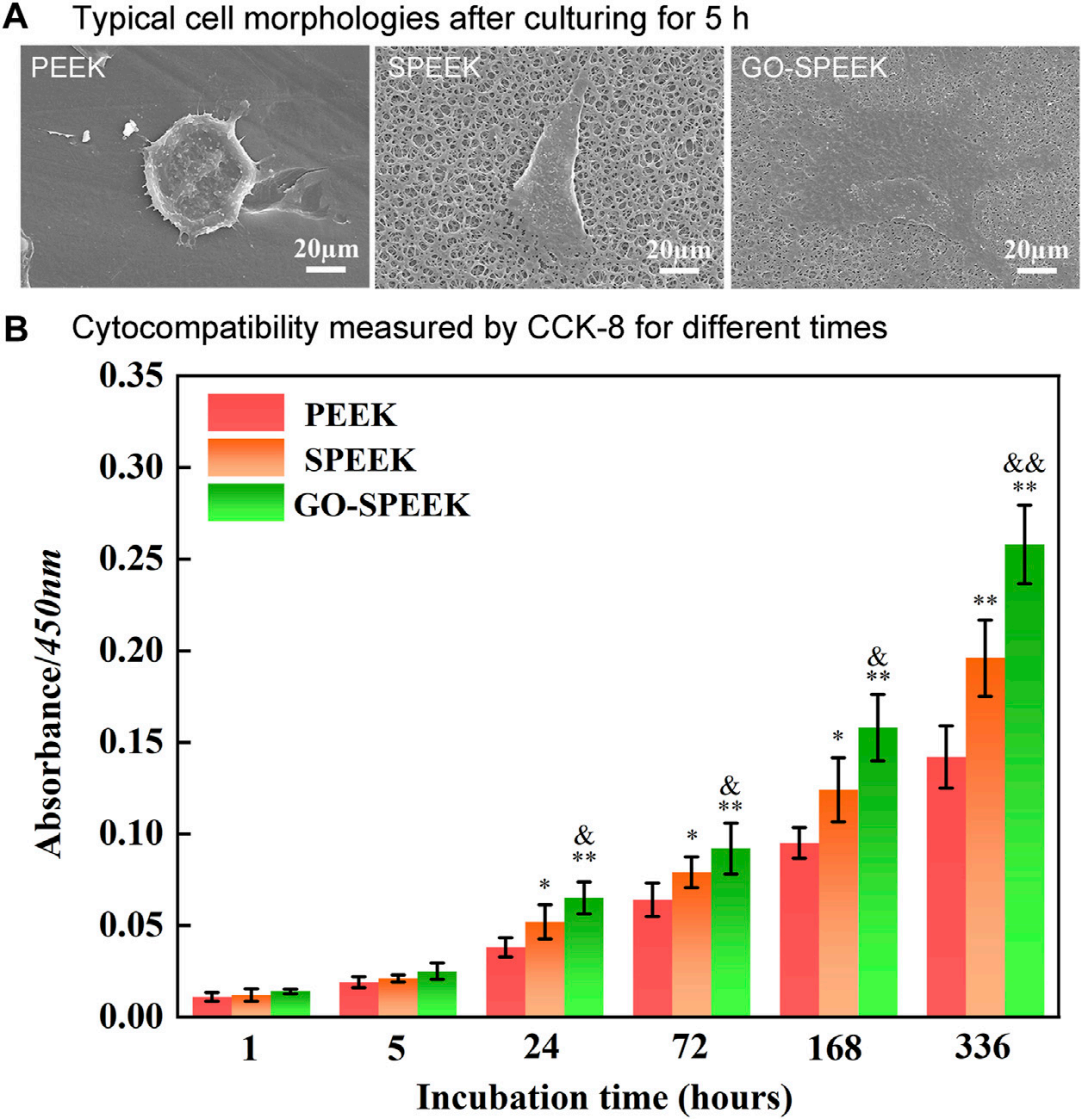
Evaluation of the cell adhesion and proliferation on the samples: **(A)** typical cell morphology on different surfaces after 5 h of incubation, **(B)** cell proliferation measured by CCK-8 after 1 h, 5 h, 24 h, 72 h, 168 h, and 336 h of culture. Data are given as the mean ± SD (*n* = 3), *p < 0.05 and **p < 0.01 compared with PEEK, &*p* < 0.05 and &&*p* < 0.01 compared with SPEEK.

In general, the adhesion of osteoblasts on the surface of a biomaterial means that osseointegration is initiated (Gongadze et al., 2011). The morphology of the MC3T3 cells after 5 h of incubation on different sample surfaces was imaged by SEM, as shown in Figure 9A. The cell is weakly adherent to the PEEK surface with an overall spherical shape, and the elongation of the cell pseudopods is not observed. In contrast, the cell on SPEEK is irregularly shaped and stretched much wider with distinct pseudopods. The cell on GO-SPEEK is observed to be fully spread and tightly bound to the surface, implying that the GO-SPEEK surface provides the optimal circumstances for the osteoblasts to attach. The CCK-8 assay was used to further disclose the adhesion and proliferation conditions of cells on the samples. It can be seen from Figure 9B that, at the initial incubation times of 1 h and 5 h, no significant difference in the viable cells adhered to the samples was observed. At 24 h, the numbers of adherent cells on the different samples all increased compared to 5 h. As time progressed, the cells on the three kinds of samples all proliferated. These results prove that the PEEK, SPEEK, and GO-PEEK samples in this study all exhibit good cytocompatibility, and they boost cell growth on the surfaces. Notably, the cell proliferation is observed to be accelerated on the SPEEK sample, and the acceleration is more pronounced on the GO-PEEK sample than on the PEEK sample.

**FIGURE 10:**
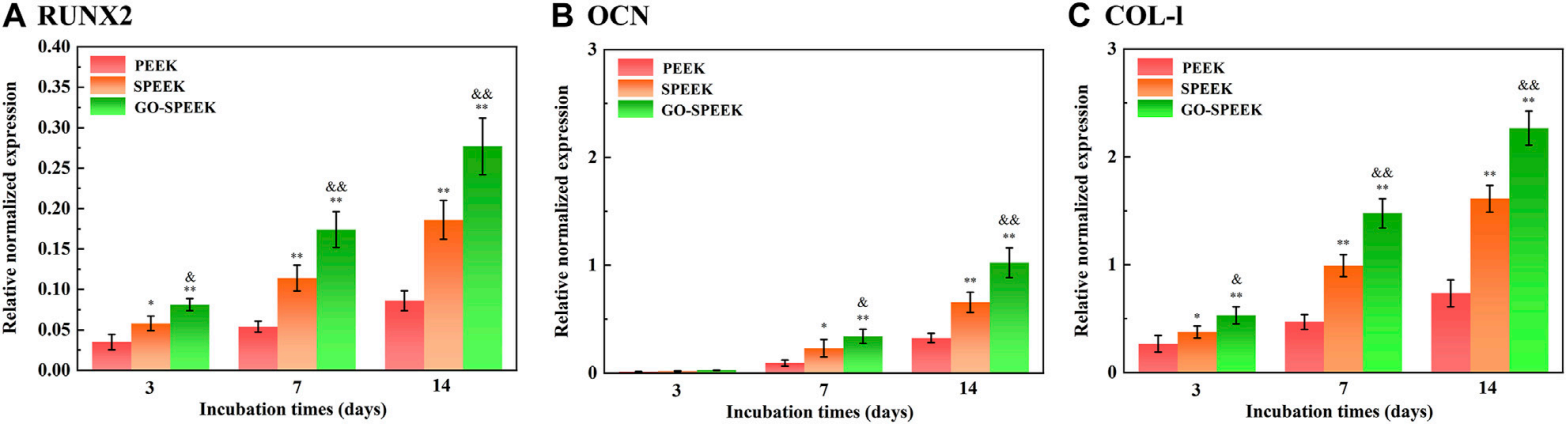
Gene expressions of cells cultured on the samples after incubation for 3 days, 7 days, and 14 days: **(A)** RUNX2, **(B)** OCN, and **(C)** Col-I. Data are given as the mean ± SD (*n* = 3), *p < 0.05 and **p < 0.01 compared with PEEK, &*p* < 0.05 and &&*p* < 0.01 compared with SPEEK.

The enhanced cell adhesion and proliferation will influence subsequent differentiation and ECM mineralization (Luo et al., 2015; Huang et al., 2021). The osteogenic differentiation of MC3T3 cells on different samples was evaluated by the determination of mRNA expression levels of several osteogenesis-related gene markers. As shown in Figure 10, the RUNX2, OCN, and Col-I mRNA expression levels of the cells on each sample sustainably increase with an incubation time of up to 14 days. At each time point, the expressions of mRNA of each marker in the cells on the SPEEK sample are higher than that on the PEEK sample. However, the GO-SPEEK sample exhibits the strongest positive effect of boosting the osteogenic differentiation of cells.

**FIGURE 11:**
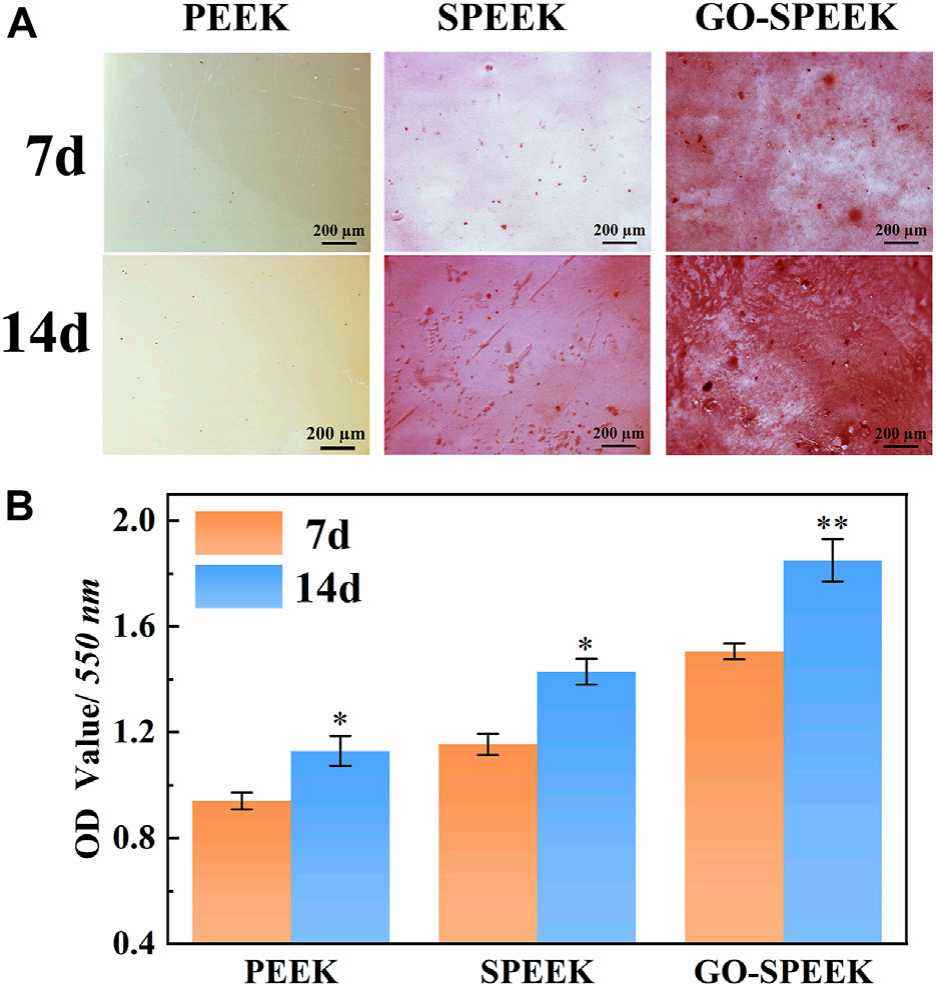
**(A)** Alizarin red staining pictures of the samples after 7 days and 14 days of incubation, and **(B)** ECM mineralization after 7 days and 14 days of osteoblast incubation on different samples. Data are given as the mean ± SD (*n* = 3), *p < 0.05 and **p < 0.01 compared with 7 days of incubation.

RUNX2 is a transcription factor necessary for early osteoblast differentiation. OCN, responsible for calcium ion binding, is a late-stage maker of osteoblast differentiation, and Col-I is the most abundant bone matrix protein (Huang et al., 2021). In this work, GO-SPEEK remarkably enhances levels of RUNX2 mRNA compared to SPEEK and PEEK (Figure 10A), indicating that GO-SPEEK can accelerate the differentiation by promoting a mature phenotype at earlier time points (Kaur et al., 2024). The upregulation of RUNX2 mRNA expression was reported to facilitate the mRNA expression of OCN and Col-I (Huang et al., 2019), thus leading to more pronounced OCN and Col-I proteins to secrete into the ECM and thereby accelerating ECM mineralization. The ECM mineralization results in our investigation confirmed the above point. As seen in Figure 11A, it is obvious that much denser ECM mineralization is deposited on the GO-SPEEK surface than on the PEEK and SPEEK surfaces (especially the PEEK surface) at each time point. In addition, at 14 days, much denser stained deposits are observed on the SPEEK and GO-SPEEK samples than at 7 days. The quantitatively determined ECM mineralization results are in accordance with the above staining pictures, as shown in Figure 11B. It is indicated that both SPEEK and GO-SPEEK reveal promote ECM mineralization compared to PEEK, and the mineralization level was much higher on the GO-SPEEK surface than on the SPEEK surface at each incubation time.

In this study, we built a 3D porous structure on PEEK through sulfonation and found the modified surface promotes the adhesion and osteogenic functions of MC3T3 cells. Additionally, GO grafted on the sulfonated PEEK surface was proven to further improve the cell–material interactions. The generated microporous structure of the implant surface has been reported to be favorable for cell adhesion and osteogenic functions (Lee et al., 2004; Liu et al., 2021). Cai et al. (2020) used a femtosecond laser to construct a porous surface on a PEEK surface, which was also proved to significantly promote the adhesion and proliferation of rat bone marrow mesenchymal stem cells. Apart from the microporous structure, GO could also provide an affirmative effect to regulate cell behavior. Su et al. (2020) combined GO with polydopamine and loaded it on the surface of a titanium alloy, which greatly enhanced the osteogenic mineralization of hMSCs. The previous study disclosed that the combination of π–π stacking, electrostatic forces, and hydrogen bonding of the GO surface allows it to take up osteoinductive factors from the medium via non-covalent binding, thus enhancing osteogenesis (Lee et al., 2011). Some studies have proposed that the GO can activate osteogenesis-related signaling pathways and promote bone formation (Wu et al., 2015; Zhang et al., 2016; Wu et al., 2018). Moreover, it is notable that, in this work, both sulfonation and GO grafting are beneficial for the enhancement of PEEK hydrophilicity (as shown in Figure 4D). The hydrophilic surfaces were confirmed to be conducive to regulating the osteoblastic response (Sun et al., 2017; Huang et al., 2019; Kopac, 2021; Tseng et al., 2021). Hence, the improvement of the hydrophilicity of PEEK after surface modification is also responsible for the ameliorated cell-material interactions (including cell adhesion, proliferation, osteodifferentiation, and ECM mineralization) in our investigation.

### 3.5 Mechanical properties

**FIGURE 12:**
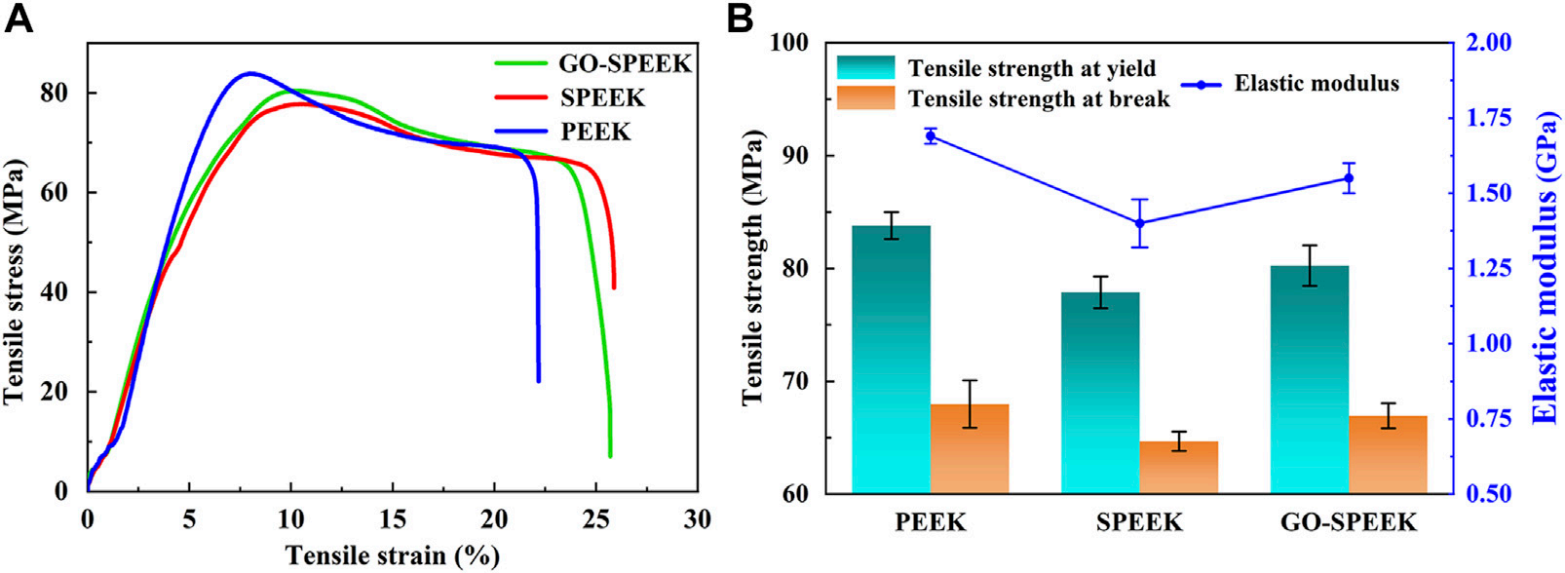
**(A)** Tensile stress *versus* tensile strain plot for PEEK, SPEEK, and GO-SPEEK; **(B)** tensile strength and elastic modulus of different samples. Data are given as the mean ± SD (*n* = 3).

The mechanical properties of the samples evaluated by using the tensile experiment and the typical tensile curve, as well as the acquired data, are shown in Figure 12. PEEK, SPEEK, and GO-SPEEK all have tensile yield zones, indicating that the samples are generally ductile materials. The tensile stress *versus* tensile strain curves of different samples substantially look analogous (Figure 12A), suggesting that little variation of the mechanical properties of PEEK occurs after the surface modification. Figure 12B compares the tensile strength and the elastic modulus of different samples, and the data are summarized in Table 2. SPEEK and GO-SPEEK have similar values of yield/break tensile strength and elastic modulus compared to the original PEEK because sulfonation and the subsequent GO-grafting treatment were only applied to the surface of the samples; the mechanical properties of the bulk material were maintained. Although the measured elastic modulus of GO-SPEEK (1.55 GPa) is slightly less than PEEK (1.69 GPa), which might be due to the formed micropores on the surface, the value is still much larger than that of the 1.33 GPa of the cancellous bone (Clausing et al., 2023). With the maintained mechanical properties, the enhanced surface biomineralization capacity, and cell-material interactions, as well as the highly efficient bacterial killing activity, GO-SPEEK is expected to be extensively used in the bone replacement and orthopedic field.

**TABLE 2:** Parameters of the mechanical properties of different samples.

Samples	Yield strength (MPa)	Break strength (MPa)	Elastic modulus (GPa)
PEEK	83.80 ± 1.22	67.98 ± 2.14	1.69 ± 0.03
SPEEK	77.89 ± 1.43	64.68 ± 0.96	1.40 ± 0.08
GO-SPEEK	80.35 ± 1.87	66.94 ± 1.15	1.55 ± 0.05

## 4 Conclusion

GO-SPEEK was fabricated using a two-step method of surface sulfonation followed by GO grafting on PEEK. The results show that the GO-SPEEK surface possesses a 3D porous structure and exhibits superior hydrophilicity to the original PEEK. In addition, GO-SPEEK reveals a good bactericidal effect against Gram-negative (*E. coli*) and Gram-positive (*S. aureus*) bacteria, and the antibacterial rate for the two kinds of bacteria strains is about 86% and 94%, respectively. GO-SPEEK displays much better biomineralization capacity than PEEK and SPEEK (especially compared to PEEK) and has the strongest activity to induce HA precipitation on the surface while soaking in an SBF solution. The *in vitro* cellular experiments indicate that osteoblastic adhesion, proliferation, osteodifferentiation, and ECM mineralization could also be promoted on the GO-SPEEK surface due to the positive effect of the hydrophilicity and the porous structure as well as the grafted GO for MC3T3 cells. Furthermore, the tensile test result suggests that GO-SPEEK has comparable values of elastic modulus and tensile strength to those of the original PEEK. With the maintained mechanical properties, enhanced surface biomineralization capacity, and cell-material interactions, as well as the highly efficient bacterial killing activity, GO-SPEEK is expected to be widely utilized in the orthopedic implantation field.

## Data Availability

The raw data supporting the conclusion of this article will be made available by the authors, without undue reservation.

## References

[B1] Akhavan O. Ghaderi E. (2010). Toxicity of graphene and graphene oxide nanowalls against bacteria. ACS Nano 4, 5731–5736. 10.1021/nn101390x 20925398

[B2] Al-Gaashani R. Najjar A. Zakaria Y. Mansour S. Atieh M. A. (2019). XPS and structural studies of high quality graphene oxide and reduced graphene oxide prepared by different chemical oxidation methods. Ceram. Int. 45, 14439–14448. 10.1016/j.ceramint.2019.04.165

[B3] Alimohammadi M. Ramazani S. A. A. (2023). Surface modification of polyether ether ketone implant with a novel nanocomposite coating containing poly (vinylidene fluoride) toward improving piezoelectric and bioactivity performance. Colloids Surf. B Biointerfaces 222, 113098. 10.1016/j.colsurfb.2022.113098 36529036

[B4] Almasi D. Izman S. Assadian M. Ghanbari M. Kadir M. R. A. (2014). Crystalline HA coating on peek via chemical deposition. Appl. Surf. Sci. 314, 1034–1040. 10.1016/j.apsusc.2014.06.074

[B5] Arciola C. R. Campoccia D. Montanaro L. (2018). Implant infections: adhesion, biofilm formation and immune evasion. Nat. Rev. Microbiol. 16, 397–409. 10.1038/s41579-018-0019-y 29720707

[B6] Barooghi B. Sheikhi M. Amiri A. (2018). Effect of nano-hydroxyapatite and dutycycle on the structure and corrosion performance of plasma electrolyte oxidation coatings in simulated body fluid on Ti-6Al-4 V. Proc. IMechE. Part C. J. Mech. Eng. Sci. 232, 4229–4236. 10.1177/0954406217752532

[B7] Basgul C. Yu T. MacDonald D. W. Siskey R. Marcolongo M. Kurtz S. M. (2018). Structure-Property relationships for 3D printed PEEK intervertebral lumbar cages produced using fused filament fabrication. J. Mat. Res. 33, 2040–2051. 10.1557/jmr.2018.178 PMC6289530 30555210

[B8] Bastan F. E. Atiq Ur Rehman M. Avcu Y. Y. Avcu E. Ustel F. Boccaccini A. R. (2018). Electrophoretic co-deposition of PEEK-hydroxyapatite composite coatings for biomedical applications. Colloids Surf. B Biointerfaces 169, 176–182. 10.1016/j.colsurfb.2018.05.005 29772473

[B9] Bhaskar N. Kachappilly M. C. Bhushan V. Pandya H. J. Basu B. (2023). Electrical field stimulated modulation of cell fate of pre-osteoblasts on PVDF/BT/MWCNT based electroactive biomaterials. J. Biomed. Mat. Res. 111, 340–353. 10.1002/jbm.a.37472 36403282

[B10] Cai G. Q. Wang H. Jung Y. K. Xu Z. Y. Zhang J. H. He J. Y. (2020). Hierarchically porous surface of PEEK/nMCS composite created by femtosecond laser and incorporation of resveratrol exhibiting antibacterial performances and osteogenic activity *in vitro* . Compos. Part B 186, 107802. 10.1016/j.compositesb.2020.107802

[B11] Cao J. Su W. Wu Z. Kitayama T. Hatada K. (1994). Synthesis and properties of poly(ether ether ketone)-poly(ether sulfone) block copolymers. Polymer 35, 3549–3556. 10.1016/0032-3861(94)90922-9

[B12] Chen Y. S. Lin J. H. C. Wu Y. R. Chang C. W. Chang K. C. Chen C. C. (2018). Characterizing the differentiation of osteoprogenitor cells on surface modified polyether-ether-ketone. Surf. Coat. Tech. 350, 904–912. 10.1016/j.surfcoat.2018.03.071

[B13] Clausing R. J. Stiller A. Kuhn F. Fonseca Ulloa C. A. Folsch C. Kampschulte M. (2023). Measuring Young's modulus of single trabeculae in cancellous bone using a two-point bending test. Clin. Biomech. (Bristol, Avon) 102, 105875. 10.1016/j.clinbiomech.2023.105875 36634601

[B14] Dallavalle M. Calvaresi M. Bottoni A. Melle-Franco M. Zerbetto F. (2015). Graphene can wreak havoc with cell membranes. ACS Appl. Mat. Interfaces. 7, 4406–4414. 10.1021/am508938u 25648559

[B15] Deng Y. Liu X. Xu A. Wang L. Luo Z. Zheng Y. (2015). Effect of surface roughness on osteogenesis *in vitro* and osseointegration *in vivo* of carbon fiber-reinforced polyetheretherketone-nanohydroxyapatite composite. Int. J. Nanomedicine 10, 1425–1447. 10.2147/ijn.s75557 25733834 PMC4337592

[B16] Dikin D. A. Stankovich S. Zimney E. J. Piner R. D. Dommett G. H. Evmenenko G. (2007). Preparation and characterization of graphene oxide paper. Nature 448, 457–460. 10.1038/nature06016 17653188

[B17] Ferrari A. C. Basko D. M. (2013). Raman spectroscopy as a versatile tool for studying the properties of graphene. Nat. Nanotechnol. 8, 235–246. 10.1038/nnano.2013.46 23552117

[B18] Gao F. Xu C. Y. Hu H. T. Wang Q. Gao Y. Y. Chen H. (2015). Biomimetic synthesis and characterization of hydroxyapatite/graphene oxide hybrid coating on Mg alloy with enhanced corrosion resistance. Mat. Lett. 138, 25–28. 10.1016/j.matlet.2014.09.088

[B19] Gongadze E. Kabaso D. Bauer S. Slivnik T. Schmuki P. van Rienen U. (2011). Adhesion of osteoblasts to a nanorough titanium implant surface. Int. J. Nanomedicine 6, 1801–1816. 10.2147/ijn.s21755 21931478 PMC3173045

[B20] Gristina A. G. (1987). Biomaterial-centered infection: microbial adhesion versus tissue integration. Science 237, 1588–1595. 10.1126/science.3629258 3629258

[B21] Guo C. Lu R. Wang X. Chen S. (2021a). Antibacterial activity, bio-compatibility and osteogenic differentiation of graphene oxide coating on 3D-network poly-ether-ether-ketone for orthopaedic implants. J. Mat. Sci. Mat. Med. 32, 135. 10.1007/s10856-021-06614-7 PMC8550550 34704134

[B22] Guo C. Lu R. Wang X. Chen S. (2021b). Graphene oxide-modified polyetheretherketone with excellent antibacterial properties and biocompatibility for implant abutment. Macromol. Res. 29, 351–359. 10.1007/s13233-021-9042-3

[B23] Hamai R. Shirosaki Y. Miyazaki T. (2018). Structural effects of sulfur-containing functional groups on apatite formation on Ca^2+^-modified copolymers in a simulated body environment. ACS Omega 3, 5627–5633. 10.1021/acsomega.8b00694 30023925 PMC6045416

[B24] He J. Zhu X. Qi Z. Wang C. Mao X. Zhu C. (2015). Killing dental pathogens using antibacterial graphene oxide. ACS Appl. Mat. Interfaces 7, 5605–5611. 10.1021/acsami.5b01069 25705785

[B25] Hemmati F. Rezaee M. A. Ebrahimzadeh S. Yousefi L. Nouri R. Kafil H. S. (2021). Novel strategies to combat bacterial biofilms. Mol. Biotechnol. 63, 569–586. 10.1007/s12033-021-00325-8 33914260

[B26] Hong J. Shah N. J. Drake A. C. DeMuth P. C. Lee J. B. Chen J. (2012). Graphene multilayers as gates for multi-week sequential release of proteins from surfaces. ACS Nano 6, 81–88. 10.1021/nn202607r 22176729 PMC4040355

[B27] Huang C. M. Chen C. H. Pornpattananangkul D. Zhang L. Chan M. Hsieh M. F. (2011). Eradication of drug resistant *Staphylococcus aureus* by liposomal oleic acids. Biomaterials 32, 214–221. 10.1016/j.biomaterials.2010.08.076 20880576 PMC2987540

[B28] Huang R. Liu L. Li B. Qin L. Huang L. Yeung K. W. K. (2021). Nanograins on Ti-25Nb-3Mo-2Sn-3Zr alloy facilitate fabricating biological surface through dual-ion implantation to concurrently modulate the osteogenic functions of mesenchymal stem cells and kill bacteria. J. Mat. Sci. Technol. 73, 31–44. 10.1016/j.jmst.2020.07.048

[B29] Huang R. Zhang L. Huang L. Zhu J. (2019). Enhanced *in-vitro* osteoblastic functions on β-type titanium alloy using surface mechanical attrition treatment. Mat. Sci. Eng. C 97, 688–697. 10.1016/j.msec.2018.12.082 30678957

[B30] Kaur G. Wu B. M. Murali S. Lanigan T. Coleman R. M. (2024). A synthetic, closed-looped gene circuit for the autonomous regulation of RUNX2 activity during chondrogenesis. FASEB J. 38, 23484. 10.1096/fj.202300348rr PMC10981937 38407380

[B31] Kiew S. F. Kiew L. V. Lee H. B. Imae T. Chung L. Y. (2016). Assessing biocompatibility of graphene oxide-based nanocarriers: a review. J. Control Release 226, 217–228. 10.1016/j.jconrel.2016.02.015 26873333

[B32] Kopac T. (2021). Protein corona, understanding the nanoparticle-protein interactions and future perspectives: a critical review. Int. J. Biol. Macromol. 169, 290–301. 10.1016/j.ijbiomac.2020.12.108 33340622

[B33] Kumar S. R. Hu C. C. Vi T. T. T. Chen D. W. Lue S. J. J. (2023). Antimicrobial peptide conjugated on graphene oxide-containing sulfonated polyetheretherketone substrate for effective antibacterial activities against staphylococcus aureus. Antibiotics 12, 1407. 10.3390/antibiotics12091407 37760704 PMC10525520

[B34] Kurtz S. M. (2019). “An overview of PEEK biomaterials,” in PEEK biomaterials handbook (London: ISBN), 3–9.

[B35] Laureto J. J. Pearce J. M. (2018). Anisotropic mechanical property variance between ASTM D638-14 type i and type iv fused filament fabricated specimens. Polym. Test. 68, 294–301. 10.1016/j.polymertesting.2018.04.029

[B36] Lee J. H. Jang H. L. Lee K. M. Baek H. R. Jin K. Hong K. S. (2013). *In vitro* and *in vivo* evaluation of the bioactivity of hydroxyapatite-coated polyetheretherketone biocomposites created by cold spray technology. Acta. Biomater. 9, 6177–6187. 10.1016/j.actbio.2012.11.030 23212079

[B37] Lee S. J. Choi J. S. Park K. S. Khang G. Lee Y. M. Lee H. B. (2004). Response of MG63 osteoblast-like cells onto polycarbonate membrane surfaces with different micropore sizes. Biomaterials 25, 4699–4707. 10.1016/j.biomaterials.2003.11.034 15120516

[B38] Lee W. C. Lim C. H. Shi H. Tang L. A. Wang Y. Lim C. T. (2011). Origin of enhanced stem cell growth and differentiation on graphene and graphene oxide. ACS Nano 5, 7334–7341. 10.1021/nn202190c 21793541

[B39] Lee W. T. Koak J. Y. Lim Y. J. Kim S. K. Kwon H. B. Kim M. J. (2012). Stress shielding and fatigue limits of poly-ether-ether-ketone dental implants. J. Biomed. Mat. Res. B Appl. Biomater. 100, 1044–1052. 10.1002/jbm.b.32669 22331553

[B40] Liu C. Qiao W. Wang C. Wang H. Zhou Y. Gu S. (2021). Effect of poly (lactic acid) porous membrane prepared via phase inversion induced by water droplets on 3T3 cell behavior. Int. J. Biol. Macromol. 183, 2205–2214. 10.1016/j.ijbiomac.2021.05.197 34087303

[B41] Liu W. Li J. Cheng M. Wang Q. Qian Y. Yeung K. W. K. (2019). A surface-engineered polyetheretherketone biomaterial implant with direct and immunoregulatory antibacterial activity against methicillin-resistant *Staphylococcus aureus* . Biomaterials 208, 8–20. 10.1016/j.biomaterials.2019.04.008 30986611

[B42] Liu X. Gan K. Liu H. Song X. Chen T. Liu C. (2017). Antibacterial properties of nano-silver coated PEEK prepared through magnetron sputtering. Dent. Mat. 33, 348–360. 10.1016/j.dental.2017.06.014 28734568

[B43] Lu P. Ma D. Chen Y. Guo Y. Chen G. Q. Deng H. (2013). L-glutamine provides acid resistance for *Escherichia coli* through enzymatic release of ammonia. Cell. Res. 23, 635–644. 10.1038/cr.2013.13 23337585 PMC3641589

[B44] Lu T. Liu X. Qian S. Cao H. Qiao Y. Mei Y. (2014). Multilevel surface engineering of nanostructured TiO_2_ on carbon-fiber-reinforced polyetheretherketone. Biomaterials 35, 5731–5740. 10.1016/j.biomaterials.2014.04.003 24767786

[B45] Luo Y. Shen H. Fang Y. Cao Y. Huang J. Zhang M. (2015). Enhanced proliferation and osteogenic differentiation of mesenchymal stem cells on graphene oxide-incorporated electrospun poly(lactic-co-glycolic acid) nanofibrous mats. ACS Appl. Mat. Interfaces 7, 6331–6339. 10.1021/acsami.5b00862 25741576

[B46] Ma T. T. Zhang J. J. Sun S. Y. Meng W. Q. Zhang Y. Wu J. L. (2023). Current treatment methods to improve the bioactivity and bonding strength of PEEK for dental application: a systematic review. Eur. Polym. J. 183, 111757. 10.1016/j.eurpolymj.2022.111757

[B47] Mahjoubi H. Buck E. Manimunda P. Farivar R. Chromik R. Murshed M. (2017). Surface phosphonation enhances hydroxyapatite coating adhesion on polyetheretherketone and its osseointegration potential. Biomater 47, 149–158. 10.1016/j.actbio.2016.10.004 27717913

[B48] Mo S. Mehrjou B. Tang K. W. Wang H. Y. Huo K. F. Qasim A. M. (2020). Dimensional-dependent antibacterial behavior on bioactive micro/nano polyetheretherketone (PEEK) arrays. Chem. Eng. J. 392, 123736. 10.1016/j.cej.2019.123736

[B49] Naskar D. Ghosh A. K. Mandal M. Das P. Nandi S. K. Kundu S. C. (2017). Dual growth factor loaded nonmulberry silk fibroin/carbon nanofiber composite 3D scaffolds for *in vitro* and *in vivo* bone regeneration. Biomaterials 136, 67–85. 10.1016/j.biomaterials.2017.05.014 28521202

[B50] Ouyang L. Deng Y. Yang L. Shi X. Dong T. Tai Y. (2018b). Graphene-oxide-decorated microporous polyetheretherketone with superior antibacterial capability and *in vitro* osteogenesis for orthopedic implant. Macromol. Biosci. 18, 1800036. 10.1002/mabi.201800036 29719124

[B51] Ouyang L. Sun Z. Wang D. Qiao Y. Zhu H. Ma X. (2018a). Smart release of doxorubicin loaded on polyetheretherketone (PEEK) surface with 3D porous structure. Colloids Surf. B Biointerfaces 163, 175–183. 10.1016/j.colsurfb.2017.12.045 29291503

[B52] Ouyang L. Zhao Y. Jin G. Lu T. Li J. Qiao Y. (2016). Influence of sulfur content on bone formation and antibacterial ability of sulfonated PEEK. Biomaterials 83, 115–126. 10.1016/j.biomaterials.2016.01.017 26773668

[B53] Popok V. N. (2019). “High-fluence ion implantation of polymers: evolution of structure and composition,” in Radiation effects in polymeric materials (Germany: Springer), 69–111.

[B54] Qian G. W. Mao Y. Q. Shuai Y. Zeng Z. K. Peng S. P. Shuai C. J. (2024a). Enhancing bone scaffold interfacial reinforcement through *in situ* growth of metal-organic frameworks (MOFs) on strontium carbonate: achieving high strength and osteoimmunomodulation. J. Colloid Interf. Sci. 655, 43–57. 10.1016/j.jcis.2023.10.133 37925968

[B55] Qian G. W. Mao Y. Q. Zhao H. H. Zhang L. M. Xiong L. Long Z. S. (2024b). pH-responsive nanoplatform synergistic gas/photothermal therapy to eliminate biofilms in poly(L-lactic acid) scaffolds. J. Mat. Chem. B 12, 1379–1392. 10.1039/d3tb02600k 38247429

[B56] Qian G. W. Zhang L. M. Shuai Y. Wu X. T. Zeng Z. K. Peng S. P. (2023). 3D-printed CuFe2O4-MXene/PLLA antibacterial tracheal scaffold against implantation-associated infection. Appl. Surf. Sci. 614, 156108. 10.1016/j.apsusc.2022.156108

[B57] Sobieraj M. C. Kurtz S. M. Rimnac C. M. (2009). Notch sensitivity of PEEK in monotonic tension. Biomaterials 30, 6485–6494. 10.1016/j.biomaterials.2009.08.020 19733391 PMC2760343

[B58] Stratton-Powell A. A. Pasko K. M. Brockett C. L. Tipper J. L. (2016). The biologic response to polyetheretherketone (PEEK) wear particles in total joint replacement: a systematic review. Clin. Orthop. Relat. Res. 474, 2394–2404. 10.1007/s11999-016-4976-z 27432420 PMC5052196

[B59] Su J. Du Z. Xiao L. Wei F. Yang Y. Li M. (2020). Graphene oxide coated titanium surfaces with osteoimmunomodulatory role to enhance osteogenesis. Mat. Sci. Eng. C 113, 110983. 10.1016/j.msec.2020.110983 32487397

[B60] Sun J. Yao Q. T. Zhang Y. H. Du X. D. Wu Y. C. Tong W. P. (2017). Simultaneously improving surface mechanical properties and *in vitro* biocompatibility of pure titanium via surface mechanical attrition treatment combined with low-temperature plasma nitriding. Surf. Coat. Tech. 309, 382–389. 10.1016/j.surfcoat.2016.11.095

[B61] Sun Z. Ouyang L. Ma X. Qiao Y. Liu X. (2018). Controllable and durable release of BMP-2-loaded 3D porous sulfonated polyetheretherketone (PEEK) for osteogenic activity enhancement. Colloids Surf. B Biointerfaces 171, 668–674. 10.1016/j.colsurfb.2018.08.012 30107340

[B62] Sutherland J. P. Bayliss A. J. Roberts T. A. (1994). Predictive modelling of growth of *Staphylococcus aureus*: the effects of temperature, pH and sodium chloride. Int. J. Food Microbiol. 21, 217–236. 10.1016/0168-1605(94)90029-9 8024974

[B63] Torrisi L. Silipigni L. Cutroneo M. Torrisi A. (2020). Graphene oxide as a radiation sensitive material for XPS dosimetry. Vaccum 173, 109175. 10.1016/j.vacuum.2020.109175

[B64] Toth J. M. Wang M. Estes B. T. Scifert J. L. Seim H. B. Turner A. S. (2006). Polyetheretherketone as a biomaterial for spinal applications. Biomaterials 27, 324–334. 10.1016/j.biomaterials.2005.07.011 16115677

[B65] Tseng S. J. Cheng C. H. Lee T. M. Lin J. C. (2021). Studies of osteoblast-like MG-63 cellular proliferation and differentiation with cyclic stretching cell culture system on biomimetic hydrophilic layers modified polydimethylsiloxane substrate. Biochem. Eng. J. 168, 107946. 10.1016/j.bej.2021.107946

[B66] Tu Y. Lv M. Xiu P. Huynh T. Zhang M. Castelli M. (2013). Destructive extraction of phospholipids from *Escherichia coli* membranes by graphene nanosheets. Nat. Nanotechnol. 8, 594–601. 10.1038/nnano.2013.125 23832191

[B67] Wang S. Yang Y. Li Y. Shi J. Zhou J. Zhang L. (2019). Strontium/adiponectin co-decoration modulates the osteogenic activity of nano-morphologic polyetheretherketone implant. Colloids Surf. B Biointerfaces 176, 38–46. 10.1016/j.colsurfb.2018.12.056 30592990

[B68] Waser-Althaus J. Salamon A. Waser M. Padeste C. Kreutzer M. Pieles U. (2014). Differentiation of human mesenchymal stem cells on plasma-treated polyetheretherketone. J. Mat. Sci. Mat. Med. 25, 515–525. 10.1007/s10856-013-5072-5 24202913

[B69] Wu C. Xia L. Han P. Xu M. Fang B. Wang J. (2015). Graphene-oxide-modified β-tricalcium phosphate bioceramics stimulate *in vitro* and *in vivo* osteogenesis. Carbon 93, 116–129. 10.1016/j.carbon.2015.04.048

[B70] Wu X. Zheng S. Ye Y. Wu Y. Lin K. Su J. (2018). Enhanced osteogenic differentiation and bone regeneration of poly(lactic-*co*-glycolic acid) by graphene*via*activation of PI3K/Akt/GSK-3β/β-catenin signal circuit. Biomater. Sci. 6, 1147–1158. 10.1039/c8bm00127h 29561031

[B71] Yang H. Wu C. Deng Y. Yang W. Z. He M. M. Zhang L. (2024). MnFe2O4/graphene oxide modified PEEK with phototherapeutic and GPx-mimetic potential for anti-bacterial treatment. Mat. Lett. 357, 135609. 10.1016/j.matlet.2023.135609

[B72] Yang S. H. Yu W. Q. Zhang J. J. Han X. Wang J. Y. Sun D. (2022). The antibacterial property of zinc oxide/graphene oxide modified porous polyetheretherketone against S. sanguinis, F. nucleatumand P. gingivalis. Biomed. Mat. 17, 025013. 10.1088/1748-605x/ac51ba 35114651

[B73] Zhang W. Chang Q. Xu L. Li G. Yang G. Ding X. (2016). Graphene oxide‐copper nanocomposite‐coated porous CaP scaffold for vascularized bone regeneration via activation of hif‐1α. Adv. Healthc. Mat. 5, 1299–1309. 10.1002/adhm.201500824 26945787

[B74] Zhao J. Liu S. A. Zhang Z. J. Wu X. J. Wu D. Gong K. (2023). Investigation of the drilling performance and residual tensile behavior of polyetherketoneketone plates. Polym. Test. 120, 107967. 10.1016/j.polymertesting.2023.107967

[B75] Zhao Y. Wong H. M. Wang W. Li P. Xu Z. Chong E. Y. (2013). Cytocompatibility, osseointegration, and bioactivity of three-dimensional porous and nanostructured network on polyetheretherketone. Biomaterials 34, 9264–9277. 10.1016/j.biomaterials.2013.08.071 24041423

